# The Malarial Germ

**Published:** 1888-01

**Authors:** 


Yours truly,

F. E. Y.

### THE "MALARIAL GERM"(?)

LETTER FROM DR. FELDER, OF CLEBURNE, TEXAS; AND MICROSCOPIC EXAMINATION OF SPORES BY DR. MENGER.

CLEBURNE, TEXAS, Dec. 14, 1887.

*Dr. Daniel*:

DEAR SIR:—According to promise, I give you a history of the discovery of what I suppose to be the MALARIAL GERM.

It was in Navarro county, Texas, during the summer of 1861, that I noticed a deposit, similar to dust, upon the leaves of the Broom Weed. This deposit increasing very rapidly, and finding it upon weeds remote from ways of travel, where could be no suspicion of dust, I was prompted to make an examination.

But having nothing to aid me, except a small pocket microscope, I came to the conclusion that it was simply mould. But if mould, why was it *invariably* found upon the *upper* side of the leaf? Why had it not formed during the previous wet spell, when the earth was saturated with water, and vegetation filled with sap?

You perceive, Doctor, that this deposit appears in dry, hot weather, *after* abundant spring rains, and disappears (for a time) after a general rainfall.

These, and similar reflections, caused me to believe that this deposit was nothing but developed spores, with which the atmosphere was filled during that season of the year; and that those spores required, for their full development, heat and moisture, both of which would be furnished by the sun and the dew, in which they were deposited.

If this hypothesis be correct, then, I thought that an atmosphere so vitiated would have a deleterious effect upon the human economy, and that each appearance of this deposit would be followed by disease, the type of which would depend upon the quantity of these spores in the atmosphere, as evidenced by the quantity of this deposit. Observation then fully convinced me of the truth of that hypothesis. And now, after an observation and an experience of more than a quarter of a century in Texas, I am fully persuaded that the cause of malaria is a spore, which rises from a dry, though previously wet soil, and is suspended in the atmosphere until washed out by a general rainfall. That the sample sent you by Dr. Osburn was deposited with the dew, upon the leaf, and developed into a visible germ by the action of the sun, there can, I think, be no possible doubt. Yours truly,

JNO. L. FELDER.

[The above is only a history of the discovery of the germ. Dr. Felder has kindly promised to furnish for publication, a paper, giving his experience in the treatment of malarial diseases, based upon the supposition that the above described substance, which he takes to be a vegetable spore, is the cause of the disturbances witnessed in ague and other malarial diseases, His theory is that the blood is *thickened* by the *accumulation* of these spores, hence the local congestion.

Below, we give the microscopic appearance and a description of the substance, by Dr. Rudolph Menger. Dr. Felder sent some oak leaves covered on the upper surface by the deposit, to Dr. Osborn, who sent them to this office for examination. At our request Dr. Menger furnishes the following description of the deposit, as it appears under the microscope.

It would seem to be a most extraordinary coincidence that this "deposit" *invariably* precedes an epidemic of *congestive fever*, as observed for twenty-five years by Dr. Felder, if it really bears no relation to the disease. Dr. Felder says he considers it the *"veritable, visible malarial germ."* Ed.]

Dr. Menger writes as follows:

MICROSCOPIC APPEARANCE.

"On scraping a part of this deposit carefully with a razor, and mounting the scraped material in glycerine, the following picture presents itself under microscopic examination: The entire slides show numerous perfectly round, black bodies, from the bottom or base of which about 8 to 12 transparent threadlike filaments project. On closer examination of these black globular bodies, which are invisible to the naked eye, they consist of a general sporangium and a number of thallus threads. A distinct network is seen in those bodies when the capsule is broken, containing numerous round and oval spores. The filaments, projecting in a straight line, are not much larger than the entire sporangium, are not jointed, and do not contain any granular matter, as is the case with many other bodies of the same species. The slides also show spores in which the thallus threads are smaller than the sporangium, and others show a beautiful network at the end of each thallus thread. Besides these main ingredients, the slides contain numerous fragments of broken filaments and spores, all sort of vegetable tissue, hair and groups of spores from the derma of the leaf, all of which can also be seen on the micro-photographs prepared from the slides under examination.

In general, these black bodies resemble the pollen of some species of flowers, but the closer distinction shows that, on account of the protruding filaments from the base of each sporangium and the firm adherence to the surface of the leaf, the same is unquestionably some vegetable parasitic growth, or germ. It is a *puculiar cryptogram fnngus*, but certainly not the common mould called *penicilium glaucum* and aspergillus glaucus found on a great variety of vegetables and fruit, etc., nor is it the oidium albicans.

In how far this fungus stands in relationship to "malaria fever" I am not able to say, and further investigations would perhaps lead to a definite conclusion; but, at any rate, the matter is of great importance, and should receive closer attention by those directly interested in this matter.

R. Menger.

